# A group of three miRNAs can act as candidate circulating biomarkers in liquid biopsies from melanoma patients

**DOI:** 10.3389/fmed.2023.1180799

**Published:** 2023-06-14

**Authors:** Eleonora De Martino, Ilaria Gandin, Eros Azzalini, Cesare Massone, Maria Antonietta Pizzichetta, Erika Giulioni, Sanja Javor, Caterina Pinzani, Claudio Conforti, Iris Zalaudek, Serena Bonin

**Affiliations:** ^1^Dermatology and Venerology Unit, Department of Medical Sciences, University of Trieste, Trieste, Italy; ^2^Biostatistics Unit, Department of Medical Sciences, University of Trieste, Trieste, Italy; ^3^Dermatology Unit and Scientific Directorate, Ospedali Galliera, Genova, Italy; ^4^Oncologic Dermatology Prevention Unit, Centro di Riferimento Oncologico (CRO), IRCCS, Aviano, Italy; ^5^Dermatology Unit, AS FO Azienda sanitaria Friuli Occidentale, Pordenone, Italy; ^6^Dermatology Unit, Ospedali Galliera, Genova, Italy; ^7^Dermatology Unit, ASU GI Azienda sanitaria universitaria integrata Giuliano Isontina, Trieste, Italy

**Keywords:** liquid biopsy, plasma, exosomes, miRNA, melanoma

## Abstract

**Background:**

Staging of melanoma and follow up after melanoma diagnosis aims at predicting risk and detecting progression or recurrence at early stage, respectively in order to timely start and/or change treatment. Tumor thickness according to Breslow, status of the sentinel node and value of the lactate dehydrogenase (LDH) are well-established prognostic markers for metastatic risk, but reliable biomarkers identifying early recurrence or candidates who may benefit best from medical treatment are still warranted. Liquid biopsy has emerged to be a suitable method for identifying biomarkers for early cancer diagnosis, prognosis, therapeutic response prediction, and patient follow-up. Liquid biopsy is a blood-based non-invasive procedure that allows analyzing circulating analytes, including extracellular vesicles.

**Methods:**

In this study we have explored the use of 7 miRNAs, namely *hsa-miR-149-3p, hsa-miR-150-5p, hsa-miR-21-5p*, hsa-miR-200c-3p, *hsa-miR-134-5p, hsa-miR-144-3p* and *hsa-miR-221-3p* in plasma exosomes to discriminate melanoma patients from controls without melanoma in a cohort of 92 individuals.

**Results and discussion:**

Our results showed that three out seven miRNAs, namely *hsa-miR-200c-3p, hsa-miR-144-3p* and *hsa-miR-221-3p* were differentially expressed in plasma-derived exosomes from melanoma patients and controls. Furthermore, the expression of the three miRNAs may be a promising ancillary tool as a melanoma biomarker, even for discriminating between nevi and melanoma.

## Introduction

The staging of melanoma aims at estimating the risk of metastases in order to define individuals who benefit from close follow-up and/or medical oncological treatment. The most potent markers to estimate the risk of metastases are tumor thickness of the primary melanoma, the status of the sentinel lymph node (SLN), and the value of lactate dehydrogenase (LDH). Therefore, a high risk for metastatic disease is associated with thicker tumors (>0.8 mm), the positivity of the SLN, and increased values of the LDH. For high-risk individuals (stages II B, C, D, and stage III), the mutational analysis of primary tumor or metastases is performed in order to offer adjuvant treatment, i.e., targeted treatment (mainly BRAF and MEK inhibitors for BRAF mutant melanoma) or immune checkpoint inhibitors (ICI).

In contrast, follow-up after melanoma diagnosis aims at detecting secondary cancers, recurrence, or progression at an early stage in order to timely initiate or change concurrent oncologic medical treatment. Follow-up includes regular clinical and radiological examinations and assessment of LDH. It has been shown that ~30–40% of metastases in patients with stages II or III are self-detected, which highlights the need to improve biomarkers for disease monitoring (1, 2). In this regard, liquid biopsy has been proven to be a suitable method for monitoring and treatment response evaluation but also for early diagnosis. Several studies in metastatic melanoma patients highlighted the clinical utility of liquid biopsy in detecting and monitoring BRAF/NRAS mutations (3–5). Nonetheless, wild-type patients currently cannot benefit from this monitoring approach. Liquid biopsy provides a source also for other circulating analytes, such as exosomes, circulant cancer cells, and microRNA (miRNA). Particular attention was paid to circulating tumoral microRNA (miRNA) based on their stability and easy methodology for detection. Recent studies reported on the molecular characterization of skin melanoma through their analysis from liquid biopsy (6–8).

Seven miRNAs, *hsa-miR-149-3p, hsa-miR-150-5p, hsa-miR-21-5p, hsa-miR-200c-3p, hsa-miR-134-5p, hsa-miR-144-3p*, and *hsa-miR-221-3p*, are of particular interest in melanoma tumorigenesis. Both *hsa-miR-21-5p* and *hsa-miR-221-3p* are onco-miRNAs, and *hsa-miR-21-5p* was found upregulated in melanoma tissues, and *in vitro* experiments have established its role in cell cycle regulation by targeting the cyclin-dependent kinase inhibitor 2C (CDKN2C) (9). On the contrary, *hsa-miR-200c-3p* and *hsa-miR-144-3p* are onco-suppressor microRNAs (10, 11), however, *hsa-miR-200c-3p* is highly expressed in serum and plasma of patients with others solid tumors (12–14). Other dysregulated microRNAs are *hsa-miR-134-5p* and *hsa-miR-150-5p*, and *hsa-miR-134-5p* is involved in the regulation of cell proliferation, apoptosis, invasion, and drug resistance; only recently, its expression was investigated in liquid biopsies from patients with melanoma (7, 15). Data on *hsa-miR-150-5p* in melanoma are contradictory as in tissues and cultured cells, it seems to act as a possible tumor suppressor (16), while it appears highly expressed in plasma of patients with melanoma (6), and *hsa-miR-149-3p* seems to be involved in the interaction between cancer cells and tumor microenvironment (17).

In this study, we investigated the abovementioned miRNAs as possible diagnostic/monitoring biomarkers in plasma extracellular vesicles of a cohort of melanoma patients and controls.

## Methods and materials

### Plasma collection

This is a multi-centric study where participants were recruited from four Italian hospitals, namely, the University Hospital of Trieste, the Galliera Hospitals of Genoa, the National Cancer Institute of Aviano (CRO), and the Santa Maria Degli Angeli Hospital of Pordenone. Written informed consent was obtained from each participant, and the study was approved by the ethical committee of the study coordinator (ID study 2937, approval protocol number CEUR-2019-PR-05 of 10/09/2019). Whole peripheral blood samples were collected in EDTA or PAXgene Blood ccfDNA tubes (Cat. No.768165; PreAnalyticX, Qiagen, Hilden, Germany) during dermatological examination, before the excision of the pigmented lesion. Therefore, during the collection of blood, the diagnosis of melanoma was suspected. Finally, blood samples were withdrawn from 92 individuals. All participating hospitals enrolled consecutive patients from 01 September 2019 through 28 February 2021. To fulfill the ISO standard for pre-analytical processes (42), blood samples were collected in EDTA only from some patients of the University Hospital of Trieste, where plasma separation and molecular tests were carried out. In that case, after blood withdrawal, EDTA tubes were transferred to the laboratory at a controlled temperature (4–8°C) within 2 h of collection. For all the other samples, in order to preserve samples' stability during the storage and transport, PAXgene Blood ccfDNA tubes were used. Blood samples collected in PAXgene Blood ccfDNA tubes were delivered at the central laboratory within 7 days from blood withdrawal. Tube storage and transportation were carried out at a controlled temperature (≤25°C) as suggested by the manufacturer. Plasma was immediately separated by centrifugation upon arrival in the laboratory.

Plasma separation was obtained by centrifugation for 15 min at 3,000x *g* at room temperature in agreement with the manufacturer's indications for PaxGene tubes and following the ISO standard for EDTA tubes (42). Briefly, EDTA tubes were centrifuged for 10 min at 1,600x *g* at 4°C, and after plasma transferal to new tubes, it was centrifuged for 10 min at 13,000x *g* at 4°C. Plasma supernatant was aliquoted into 1 ml vial and stored at −80°C until use.

### RNA isolation from exosomes and extracellular vesicles

Total RNA from exosomes and extracellular vesicles (EVs) was isolated from 2 ml of plasma using the ExoRNeasy Maxi Kit (Cat. No.77164; Qiagen, Hilden, Germany) following the manufacturer's instructions. Before starting the procedure, the frozen plasma was incubated at room temperature for slow thawing, and then centrifugation of 5 min at 4°C at 3,000x *g* was performed to eliminate cryoprecipitates and residual cellular material. The standard protocol described in the *ExoRNeasy Serum/Plasma Handbook* has been followed (18). After centrifugation, plasma was diluted in a ratio of 1:1 with the binding buffer provided by the manufacturer and added to the membrane affinity column that binds exosomes and EVs to the membrane. Steps of centrifugation and washing were performed to eliminate non-specific retained material. The bound vesicles were then lysed and eluted with QIAzol. As a control, after the Qiazol elution step, 1 μl of spike-in mix (UniSp2, UniSp4, and UniSp5; Cat. No. 339390; Qiagen, Hilden, Germany) was added to the solution and incubated at room temperature for 5 min. Chloroform was added to obtain aqueous and organic phase separation. Subsequently, the aqueous phase was added to the spin column, and total RNA was eluted in 14 μl of RNase-free water (2 μl were of dead volume), split into aliquots, and stored at −80°C. The RNA quantity and purity were checked through a NanoDrop^TM^ ND-1000 spectrophotometer (Thermo Fischer Scientific, Waltham MA 02451, USA). Total RNA yield was calculated by multiplying the concentration by the elution volume (12 μl).

### Hemolysis

The impact of hemolysis was evaluated by measuring the absorbance at 414 nm and 375 nm using a NanoDrop^TM^ ND-1000 spectrophotometer (Thermo Fischer Scientific, Waltham MA 02451, USA), and the ratio between the two absorbances was calculated. Samples with a ratio less than or equal to two were considered free from hemolysis (19).

### Reverse transcription and real-time PCR setting up

Both the reverse transcription and the real-time PCR reactions were set up as reported in the Supplementary file (Supplementary Table S2).

### Reverse transcription and real-time PCR of microRNA

In total, 3 μl of total RNA were reverse transcribed using a miRCURY LNA RT kit (Qiagen, Hilden, Germany; Cat. No. 339340) following the manufacturer's protocol. During the setting up of the mix, 0.5 μl of spike-in mix (UniSp6 and cel-miR-39-3p) was added to the reaction solution.

The real-time PCR has been carried out with 40-fold diluted cDNA (40X), Fast EvaGreen qPCR Master Mix 2X (Biotium, Fremont CA 94538, USA; Cat. No. 31003), and 1 μl of specific pre-designed and validated miRCURY LNA miRNA PCR assays (QIAGEN, Hilden, Germany; Cat. No. 339306) in a total reaction volume of 10 μl. Each reaction was run in duplicate in a CFX96 Touch real-time PCR Detection system (Bio-Rad, Hercules CA, USA), using the following cycling conditions: 95°C for 10 min, 45 cycles of 95°C/10 s, and 55°C or 52°C/1 min; in addition, the melting curve was carried out to evaluate the specificity of amplified products (Table 1).

**Table 1:** microRNAs information: microRNA sequences, ID accession number, and annealing temperature.

**Name**	**Accession ID**	**Sequence**	**Annealing temperature**
*hsa-miR-191-5p*	MIMAT0000440	5′CAACGGAAUCCCAAAAGCAGCUG	55°C
*hsa-miR-24-3p*	MIMAT0000080	5′UGGCUCAGUUCAGCAGGAACAG	55°C
*hsa-miR-149-3p*	MIMAT0004609	5′AGGGAGGGACGGGGGCUGUGC	52°C
*hsa-miR-150-5p*	MIMAT0000451	5′UCUCCCAACCCUUGUACCAGUG	52°C
*hsa-miR-221-3p*	MIMAT0000278	5′AGCUACAUUGUCUGCUGGGUUUC	55°C
*hsa-miR-200c-3p*	MIMAT0000617	5′UAAUACUGCCGGGUAAUGAUGGA	55°C
*hsa-miR-134-5p*	MIMAT0000447	5′UGUGACUGGUUGACCAGAGGGG	55°C
*hsa-miR-21-5p*	MIMAT0000076	5′UAGCUUAUCAGACUGAUGUUGA	52°C
*hsa-miR-144-3p*	MIMAT0000436	5′UACAGUAUAGAUGAUGUACU	55°C

For relative quantification, the “ΔΔCt” method was used (20), where, as normalizer, the geometric mean of *hsa*-*miR-191-5p* and *hsa-miR-24-3p* was employed; and as a calibrator, a pool of cDNA made of 12 melanoma and 12 healthy samples was used. In four cases, no expression was detected for *hsa-miR-221-3p* even after repeating the analysis. In order to include those cases in the statistical analysis for *hsa-miR-221-3p*, their Ct value was set to 45, representing the amplification cycle number in the corresponding protocol, namely, the detection threshold.

### Statistical analysis

In total, two individuals were excluded from the analysis for the high missing rate in microRNA levels (>30%). Individuals without real-time PCR output were excluded from data analysis (1 case for *hsa-miR-150-5p*, 1 control, and 1 case for *hsa-miR-134-5p*). Data distribution was checked by the Shapiro–Wilk normality test, and the parametric or non-parametric tests were used for statistical analysis. Given the setting of multiple testing, the significance level was set to α = 0.05/7 = 7e – 3 (Bonferroni correction).

The diagnostic power was calculated by ROC analyses (confidence interval for AUC was calculated using the Delong method), the optimal cutoff values were obtained by the Youden index (21), and the Swets classification was used to describe the area under the curve (AUC) (22). For combined analysis, z-scores for miRNAs were calculated and used to obtain the average value. Measures of performance relative to the optimal cutoff (sensitivity, specificity, and accuracy) were internally validated. Since the performance measured in the data used to obtain the classifier will be higher than what we would obtain in new observations, leading to an optimistically biased evaluation, such “optimism” was estimated with the bootstrapping technique (23), and all measures were reported after subtracting this value. Statistical analyses were carried out using GraphPad Prism 8.0 software (San Diego, CA 92108, USA) and R 4.2.1 environment.

## Results

In this prospective multicenter study, blood samples for miRNA profiling were obtained from consecutive patients with the clinical and dermoscopic suspected diagnosis of melanoma and a control group without melanoma. Seven miRNAs were profiled by quantitative real-time PCR (qPCR) in plasma from patients with malignant melanoma (MM) and patients without melanoma (woM).

### Clinical features

In total, 44 patients with histologically confirmed malignant melanoma (MM) and 41 patients without melanoma (woM) were gender and age-matched. Of the 92 blood specimens, 7 were excluded from the control group (at collection 48 individuals) because of a previous diagnosis of melanoma. The clinical characteristics of all subjects are reported in Table 2.

**Table 2:** Clinical features of melanoma patients and controls.

**Dataset characteristics**				
*CASES*	All		44	(52%)
	Gender *N* (%)	Female	16	(36%)
		Male	28	(64%)
	Stage *N* (%)	I–II	26	(59%)
		III–IV	18	(41%)
	Ulceration status *N* (%)	Present	24	(54%)
		Absent	13	(29%)
		Unknown	7	(16%)
	Lymphocyte infiltrate brisk *N* (%)	Positive	4	(9%)
		Negative	23	(52%)
		Unknown	17	(39%)
	Age mean (SD)	[Years]	69	(11)
	Breslow's thickness mean (SD)	[mm]	3.7	(3.8)
	Hospital *N* (%)	TS	27	(61%)
		GE	3	(7%)
		CRO	2	(4%)
		PN	12	(27%)
*CONTROLS*	All		41	(48%)
	Gender *N* (%)	Female	16	(39%)
		Male	25	(61%)
	Diagnosis *N* (%)	Nevus	22	(54%)
		Basal cell carcinoma	10	(24%)
		Histiocytoma	2	(5%)
		Capillary hemangioma	1	(2%)
		Venous lake	1	(2%)
		Seborrheic keratosis	2	(5%)
		Unknown	3	(7%)
	Age mean (SD)	[years]	66	(12)
	Hospital *N* (%)	TS	1	(2%)
		GE	33	(80%)
		CRO	2	(5%)
		PN	5	(12%)
Total			85	100%

In detail, the mean age of patients with malignant melanoma was 69 years (SD = 11), while in the control group, the average age was 66 years (SD = 12, *p* = 0.1). The cases group consisted of 16 women (36%) and 28 men (64%), whereas 16 women (39%) and 25 men (61%) comprised the control group (*p* = 1.0).

At the time of blood collection, 26 (59%) patients were in stage I–II, and 18 (41%) were in stage III–IV of the disease. Patients' age was not correlated with the tumor's stage (*p* = 0.5, rho = 0.11). Among controls, most patients (54%) had a diagnosis of nevus (blue nevus, junctional nevus, and dysplastic nevus).

### Hemolysis

The sample's hemolysis was evaluated by the A_414_/A_375_ ratio. On average, the ratio did not exceed the critical value of 2, but a slight difference was measured between the cases and control groups (1.9 ± 0.5 and 1.7 ± 0.5, *p* = 0.01, respectively). The A_414_/A_375_ ratio did not vary with respect to the collection tubes (*p* = 0.08) and hospital pre-analytical processes (*p* = 0.4).

### Total RNA yield and purity

Total RNA amount and purity were investigated by a NanoDrop^TM^ ND-1000 spectrophotometer (Thermo Fischer Scientific, Waltham MA 02451, USA). No differences were found between cases and control groups in terms of both RNA yield and purity, as reported in the Supplementary file (Supplementary Table S1).

### microRNA expression and patients' features

The correlation between microRNAs fold change and patients' features was investigated. Overall, our results suggested that miRNAs expression was not affected by gender although *hsa-miR-150-5p* expression was positively correlated with patients' age (rho = 0.33, *p* = 8 −4).

### The microRNA expression and clinical features

**Figure 1:**
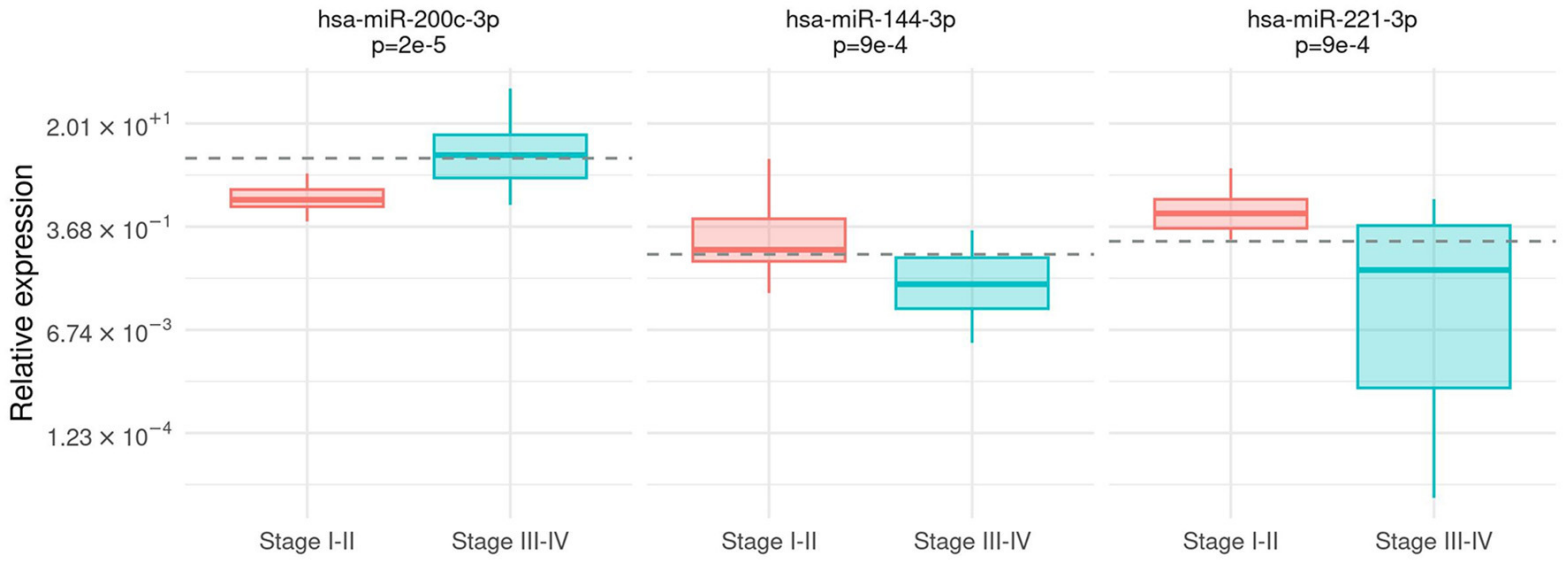
Association between tumor stage and microRNA expression. Box-plot of relative expression (log-scale) for *hsa-miR-200c-3p, hsa-miR-144-3p*, and *hsa-miR-221-3p* separately for I–II and III–IV stages. The dashed line shows the cutoff on the expression level calculated by the Youden index.

The relationship between microRNA expression and clinical features, namely, the melanoma stage, Breslow's thickness, the ulceration status, and the presence of brisk lymphocyte infiltrate, was investigated. An association between *hsa-miR-200c-3p* expression and the tumor stage was detected with significantly higher expression levels in patients with stages III and IV melanomas (Mann–Whitney test, *p* =2e−5). Contrarily, *hsa-miR-144-3p* and *hsa-miR-221-3p* were over-expressed in patients with stages I and II melanomas (Mann–Whitney test, *p* = 9e – 4 and *p* = 9e – 4, respectively) compared to higher stages (Figure 1). These three markers showed good performance in discriminating patients with stages I and II from patients with stages III and IV: AUC was 0.86 (95% CI [0.74,0.97]) for *hsa-miR-200c-3p*, 0.79 (95% CI [0.65,0.93]) for *hsa-miR-144-3p*, and 0.80 (95% CI [0.66,0.93]) for *hsa-miR-221-3p*.

### The microRNA expression in cases and control groups

**Figure 2:**
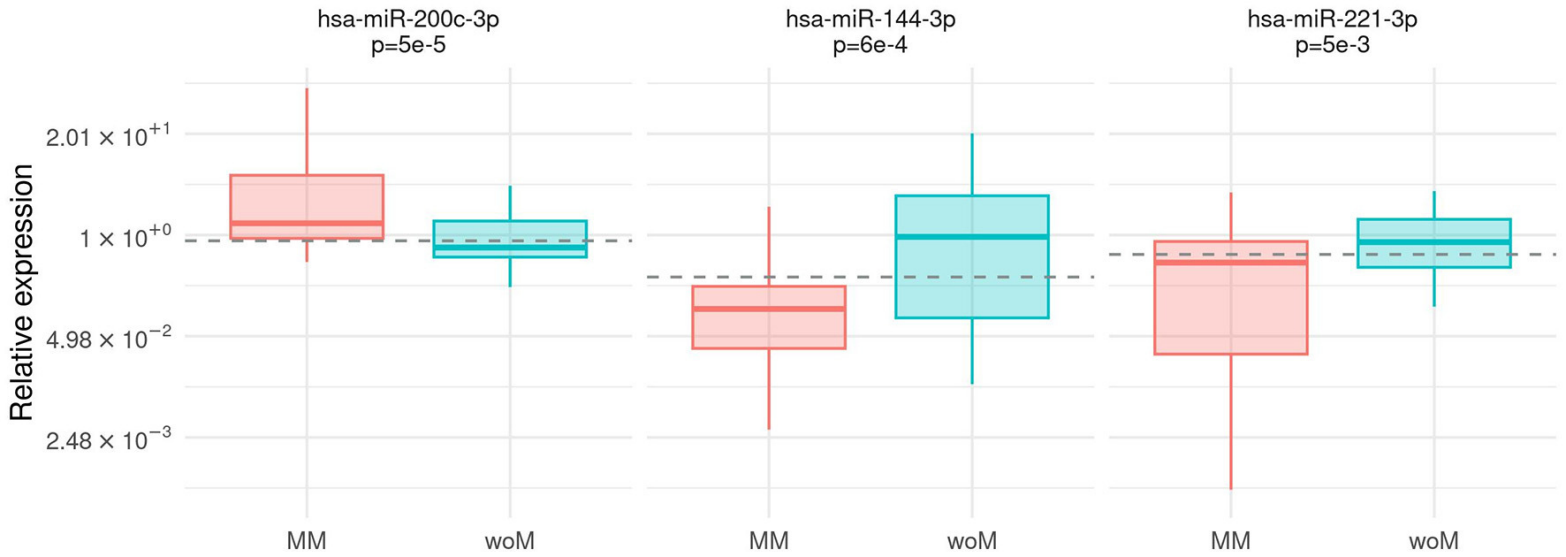
Association between case/control status and microRNA expression. Box-plot of relative expression (log-scale) of *hsa-miR-200c-3p, hsa-miR-144-3p*, and *hsa-miR-221-3* separately for malignant melanoma patients (MM) and patients without melanoma (woM). The dashed line shows the cutoff on the expression level calculated by the Youden index.

The expression of all microRNAs was compared between the group of patients with malignant melanoma (MM) and the control group (woM). The *hsa-miR-200c-3p* expression prevailed in plasma from MM (Mann–Whitney test, *p* = 5e – 5). On the contrary, *hsa-miR-144-3p* and *hsa-miR-221-3p* were significantly highly expressed in controls (Mann–Whitney test, *p* = 6e – 4, *p* = 5e – 3, respectively) (Figure 2). The association was confirmed using the multivariable analysis including age and gender as covariates (Supplementary Table S4). The diagnostic power of the abovementioned microRNAs was calculated by a ROC analysis (Supplementary Table S3). Considering the discrimination ability, *hsa-miR-221-3p* and *hsa-miR-144-3p* resulted only moderately accurate (AUC values 0.68, 95% CI [0.56, 0.79] and 0.71, 95% CI [0.60, 0.83], respectively), whereas *hsa-miR-200c-3p* could be considered a fair accurate marker (AUC value 0.75, 95% CI [0.64, 0.85]).

### The microRNA expression in nevi and melanoma

For clinicians, the differential diagnosis between melanomas and nevi could be challenging. Therefore, the fold change obtained from nevi was compared to the fold change of samples with melanoma, stratified by stages.

**Figure 3:**
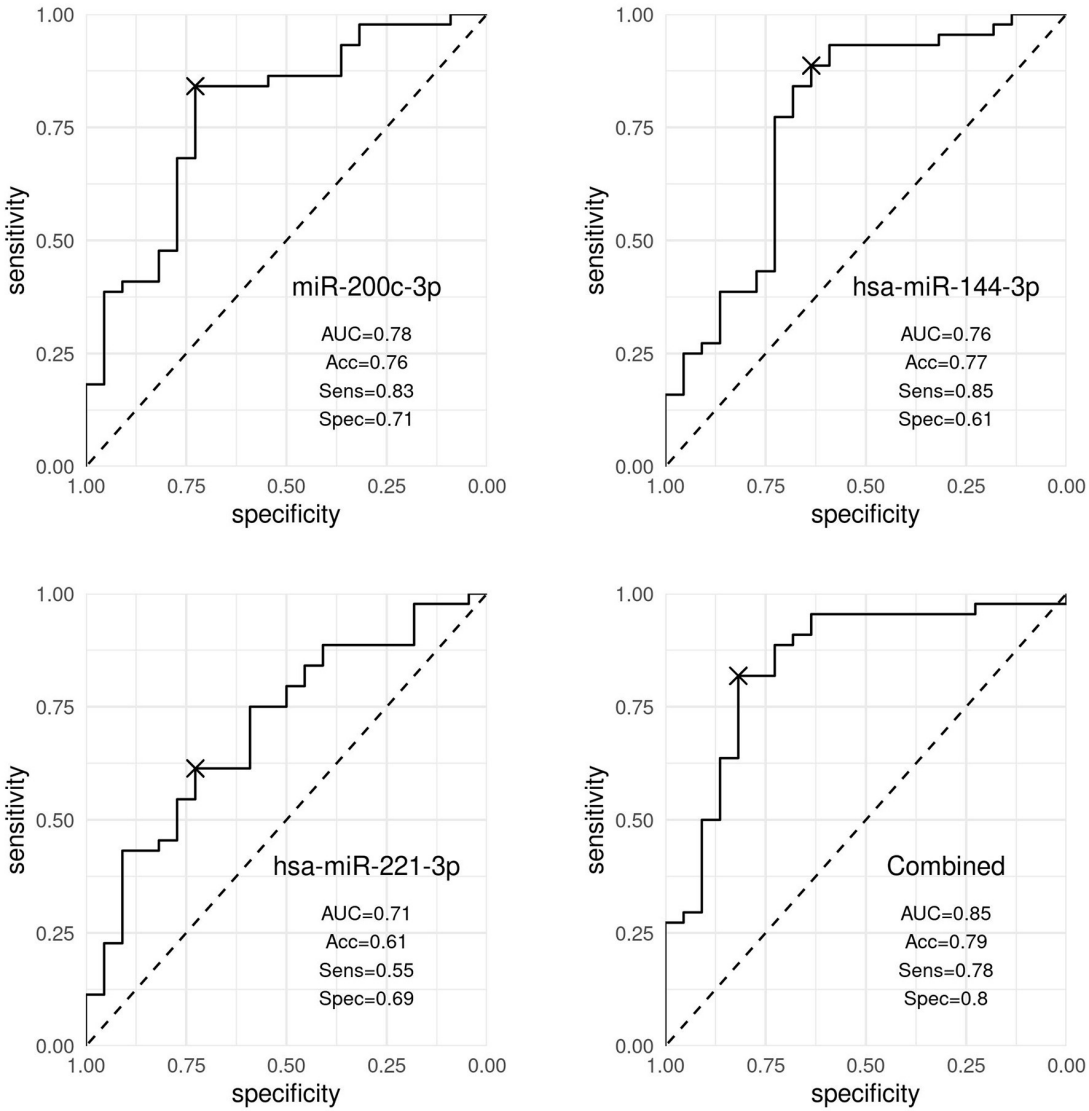
Discrimination analysis. ROC curves representing the discrimination ability of *hsa-miR-200c-3p, hsa-miR-144-3p*, and *hsa-miR-221-3p*, and the three markers expression combined, distinguishing patients with nevi and patients with melanoma. Accuracy (Acc), sensitivity (Sens), and specificity (Spec) values were obtained using the optimal cutoff point by the Youden index and corrected by optimism through internal validation.

In particular, *hsa-miR-200c-3p* accurately discriminated patients with nevi from patients with melanoma (AUC 0.78, 95% CI [0.66, 0.90]), whereas *hsa-miR-144-3p* resulted to be moderately accurate in the discrimination (AUC 0.76, 95% CI [0.63, 0.90]). Our results showed that *hsa-miR-221-3p* was a poor discriminator (AUC 0.71, 95% CI [0.54, 0.82]). By combining the three markers (*hsa-miR-200c-3p, hsa-miR-144-3p*, and *hsa-miR-221-3p*), the best discrimination was obtained (AUC 0.84, 95% CI [0.71, 0.95]), as shown in Figure 3.

## Discussion

In this study, we investigated miRNA profiles of plasma-derived exosomes from melanoma patients and controls. Three out of seven miRNAs, namely, *hsa-miR-200c-3p, hsa-miR-144-3p*, and *hsa-miR-221-3p*, were differentially expressed in plasma-derived exosomes from melanoma patients and controls. We showed that those miRNAs are differentially expressed in cases compared to controls but also in patients with early vs. advanced melanoma stages. Among miRNAs, *hsa-miR-200c-3p* was highly expressed in the plasma of melanoma patients. Moreover, it was significantly more expressed in stage III-IV melanoma samples in comparison to stage I-II. This miRNA is a member of the miR-200 family, which has a critical role in the epithelial-mesenchymal transition (EMT) in cancers (14). As a circulating biomarker, *hsa-miR-200c-3p* was increased in the plasma of metastatic breast cancer patients (14), and it was associated with progression-free survival events and reduced overall survival (24). Nevertheless, the results on *hsa-miR-200c-3p* are contradictory even in melanoma studies, where it has been reported as a tumor suppressor miRNA in tissues (25). Our results of a high expression of *hsa-miR-200c-3p*, especially in high stages of melanoma, confirm previous findings in other solid tumors (14). Notwithstanding the downregulation of *hsa-miR-200c-3p* in primary melanoma tissues has been associated with shorter survival and its expression was found higher in primary tumors than in melanoma metastases (26). Interestingly, *hsa-miR-200c-3p* upregulation in melanoma cells has been shown to lead to a rounded mode of invasion (27). In agreement with others, our results highlighted that the regulation and activity of the miR-200 family, including *hsa-miR-200c-3p*, is highly context-dependent (27). Micro RNAs, as per definition, are pleiotropic regulators with possible conflictual functions depending on the tumor microenvironment and involved cell type (28). Our results on *hsa-miR-200c-3p* can find an explanation for the well-known activity of the miR200 family as regulators of the epithelial mesenchymal transition. Taken that low miR-200 levels favor EMT, higher levels promote the mesenchymal epithelial transition, which permits cells that have escaped the primary tumor to establish colonies in a new location (14). This fits well with exosomes' function as active mediators of intercellular communication by transporting and protecting their cargoes. In our study, higher *hsa-miR-200c-3p* in circulating exosomes can be explained by the presence of circulating melanoma cells that try to seed other tissues and are prone to mesenchymal epithelial transition (MET), and this is more likely seen in advanced melanomas (stages III and IV), as shown in our results. A predicted target of *hsa-miR-200c-3p* is the microtubule-associated protein MAP2 (see Supplementary material), which seems to act as an inhibitor of melanoma cell proliferation, invasion, and tumor growth (29). MAP2 is mainly involved in dendritic morphology and has already been reported as a good prognostic marker in melanoma (30). The MAP2 activity in melanoma has also been supported by the gene expression profiling analysis (http://gepia2.cancer-pku.cn/#index), as shown in the Supplementary file of results. In detail, MAP2 resulted in significantly higher expression in the normal skin tissue when compared to melanoma. According to our results, higher *hsa-miR-200c-3p* levels in melanoma vs. controls and in higher stage melanoma support for a repression of MAP2 expression.

Both *hsa-miR-144-3p* and *hsa-miR-221-3p* expression levels are significantly lower in melanoma patients than in controls and, contrary to *hsa-miR-200c-3p*, they also resulted in significantly higher expression in low stages of melanoma compared to advanced melanoma. In uveal melanomas, miR144 was shown to act as tumor suppressor miRNA through ADAM10 and c-Met modulation (31). A decrease in *hsa-miR-144-3p* has already been reported in plasmatic levels in RCC patients with more advanced clinical-stage tumors, which also supports our findings (32). In melanoma cells, *hsa-miR-144-3p* has been shown to inhibit cell migration, supporting its tumor suppressor role (33).

It should be noted that the low *hsa-miR-221-3p* expression in high-stage melanoma patients in our study contrasts relatively with previous reports, showing a high expression of *hsa-miR-221-3p* in plasma samples of cancer patients with lung adenocarcinomas (34) and hepatocellular carcinomas (35). However, with regard to melanoma, Pfeffer et al. (36) were not able to detect any difference in the plasma levels of *hsa-miR-221-3p* between melanoma patients and healthy controls. In the serum of melanoma patients, Li et al. (37) identified miR-221 as a predictor of poor outcomes in melanoma patients, but recently other authors did not find significant differences in *hsa-miR-221-3p* levels in serum between healthy donors and melanoma patients (38). Similarly, Gasparello et al. (39) found that *hsa-miR-221-3p* did not discriminate between early CRC patients and healthy donors. In agreement with our findings, Deng et al. found that high *hsa-miR-221-3p* expression was associated with better 5-year disease-free survival of triple-negative breast cancer patients (40), and in uveal melanoma, Vashishtha et al. (41) found that the downregulation of *hsa-miR-221-3p* was associated with metastatic disease. Some reports on melanoma and *hsa-miR-221* did not specify whether−5p or−3p species were analyzed (37). Discrepancies on miR-221 can find indeed an explanation by target differences between 5p/3p species and sample differences (tissue vs. plasma). Overall, *hsa-miR-221-3p* and *hsa-miR-221-5p* seem to act mostly on different targets (by miRDIP and miDB), which explains our findings.

In conclusion, our data show that the combined expression level of three miRNAs, namely, *hsa-miR-200c-3p, hsa-miR-144-3p*, and *hsa-miR-221-3p*, is a strong candidate biomarker for discriminating between nevi and melanoma with high accuracy. If validated, this finding has possible implications both for early diagnosis and follow-up procedures. Taking the association with the melanoma stage, it is more likely that *hsa-miR-144-3p* and *hsa-miR-221-3p* could be relevant for early diagnosis while *hsa-miR-200c-3p* for follow-up monitoring of high-risk stages. We acknowledge that the main limitation of our study is the lack of an external validation cohort and that no target mRNAs were analyzed to support our hypotheses. Furthermore, although hemolysis analysis did not return any significant difference with respect to tubes and pre-analytical processes, we acknowledge an inhomogeneity in the collection of cases and controls among participant centers, without excluding a possible impact on results. Therefore, a larger and independent series of samples are needed to validate our results and to inspect the real clinical value of those miRNAs in melanoma diagnosis and follow-up.

## Data availability statement

The raw data supporting the conclusions of this article will be made available by the authors, without undue reservation.

## Ethics statement

The studies involving human participants were reviewed and approved by CEUR; ID study 2937, approval protocol number CEUR-2019-PR-05 of 10/09/2019. The patients/participants provided their written informed consent to participate in this study.

## Author contributions

IG: statistical analysis. ED, EA, and SB: formal analysis. IZ, SB, and IG: funding acquisition. ED, EA, CM, EG, CC, SJ, CP, IZ, and MP: investigation. SB and ED: methodology. ED, IG, EA, SB, CC, CM, EG, SJ, CP, MP, and IZ: resources. IZ and SB: supervision. SB, ED, EA, IG, and IZ: original draft preparation. ED, IG, EA, SB, CM, EG, CC, SJ, CP, MP, and IZ: review and editing. All authors contributed to the article and approved the submitted version.
