# Supplementary material for: A group of three miRNAs can act as candidate circulating biomarkers in liquid biopsies from melanoma patients
Source: Front Med (Lausanne). 2023 Jun 14;10:1180799. doi: 10.3389/fmed.2023.1180799 (PMC10301821; doi:10.3389/fmed.2023.1180799)
Supplement: Supplementary file 1 [file Table_1.DOCX]

## **Methods and materials**

### Reverse Transcription and Real-Time PCR setting up

In order to evaluate the appropriate volume of RNA for reverse transcription (RT), different total RNA volumes were reverse transcribed using miRCURY LNA RT kit (Qiagen, Hilden, Germany; Cat. No. 339340) following the manufacture’s protocol. One, two and three microliters were reverse transcribed with 0.5 µl of Spike-in mix in a final volume of 10 µl. Subsequently, a 40-fold dilution was amplified by Real Time PCR for Spike-ins analysis.

The Real-Time PCR efficiency was evaluated creating a standard curve with five points of serial dilutions using the pool cDNA. The points were prepared with a 1:2 serial dilution, namely the miRNA pool was diluted 40-fold (40X), 80-fold (80X), 160-fold (160X), 320-fold (320X) and 640-fold (640X).

For each microRNA the best annealing temperature was established with a gradient temperature, meaning 60°C, 55°C and 52°C.

## **Results**

### Total RNA yield and purity

Total RNA amount and purity were investigated by NanoDrop^TM^ ND-1000 spectrophotometer (Thermo Fischer Scientific, Waltham MA 02451, USA). No significant differences were reported between the cases and control groups both for total RNA yield and purity (Table S1).

In detail, the mean RNA yield of cases was of 330±222 ng and 410±278 ng was obtained from the extraction of total RNA from control samples (*p*= 0.08).

Both A260/280 and A260/230 ratios were lower than the optimal values (ratio of 2.0 for both ratios), but they are similar for the two groups (*p*= 0.8 and *p*= 0.2, in order).

|  | Total RNA yield mean±SD | *p* value ^1^ | A 260/280 mean±SD | *p* value ^1^ | A 260/230 mean±SD | *p* value ^1^ |
| --- | --- | --- | --- | --- | --- | --- |
| *CASES* | 330±222 ng | 0.08 | 1.4±0.3 | 0.8 | 0.6±0.5 | 0.2 |
| *CONTROLS* | 410±278 ng |  | 1.4±0.3 |  | 0.6±0.3 |  |
| ^1^ Mann-Whitney test | | | | | | |

**Table S1: Total RNA yield and purity.** The values are reported as mean±SD and the *p* vale refers to Mann-Whitney test.

### Real-time PCR efficiency

The Real-Time efficiency for each microRNA was obtained from 1:2 serial dilution of a pool cDNA. All efficiency results were reported in Table S2. On average, the efficiency was good for all amplifications with a mean of 92% (80-110%). Since all regression lines were parallel, the 2^-∆∆Ct method was applied.

| **Name** | **Slope** | **R^2^** | **Y-intercept** | **E** | ***p* value ^1^** |
| --- | --- | --- | --- | --- | --- |
| *hsa-miR-191-5p* | -3.6 | 0.99 | 24.8 | 0.9 | <0.0001 |
| *hsa-miR-24-3p* | -3.6 | 0.99 | 24.6 | 0.9 |  |
| *hsa-miR-149-3p* | -3.2 | 0.99 | 31.1 | 1.0 |  |
| *hsa-miR-150-5p* | -3.9 | 0.99 | 25.0 | 0.8 |  |
| *hsa-miR-221-3p* | -3.9 | 0.99 | 27.8 | 0.8 |  |
| *hsa-miR-200c-3p* | -3.1 | 0.98 | 32.2 | 1.1 |  |
| *hsa-miR-134-5p* | -3.2 | 0.93 | 33.0 | 1.0 |  |
| *hsa-miR-21-5p* | -3.6 | 0.99 | 23.6 | 0.9 |  |
| *hsa-miR-144-3p* | -3.6 | 0.99 | 22.7 | 0.9 |  |
| ^1^ Ancova test | | | | | |

**Table S2: Real-time PCR efficiency of microRNA amplification.** The *p* value refers to ANCOVA test

### The microRNA expression in cases and control groups

The diagnostic power of *hsa-miR-200c-3p,* *hsa-miR-144-3p* and *hsa-miR-221-3p* was investigated by ROC analyses (Table S3).

|  | *hsa-miR-200c-3p* | | *hsa-miR-144-3p* | | *hsa-miR-221-3p* | |
| --- | --- | --- | --- | --- | --- | --- |
| ‍ | Raw | optimism-corrected | Raw | optimism-corrected | Raw | optimism-corrected |
| Sensitivity | 0.66 [0.51,0.80] | 0.65 | 0.66 [0.52,0.80] | 0.64 | 0.68 [0.54,0.83] | 0.64 |
| Specificity | 0.84 [0.73,93] | 0.82 | 0.77 [0.64,0.89] | 0.74 | 0.61 [0.48,0.75] | 0.56 |
| Positive predicted value | 0.79 [0.68,91] | 0.78 | 0.73 [0.62,0.85] | 0.70 | 0.62 [0.52,0.73] | 0.58 |
| Negative predicted value | 0.72 [0.64,0.82] | 0.71 | 0.71 [0.62,0.81] | 0.68 | 0.767 [0.57,0.79] | 0.62 |
| Accuracy | 0.75 [0.67,0.85] | 0.74 | 0.72 [0.64,0.86] | 0.69 | 0.65 [0.54,0.74] | 0.60 |

**Table S3: ROC analyses results.** In table are reported sensitivity, specificity, positive predicted value, negative predicted value, accuracy, with their 95% interval of confidence and optimism-corrected estimate.

|  | *hsa-miR-200c-3p* | | | *hsa-miR-144-3p* | | | *hsa-miR-221-3p* | | |
| --- | --- | --- | --- | --- | --- | --- | --- | --- | --- |
|  | OR | 95%CI | p-value | OR | 95%CI | p-value | OR | 95%CI | p-value |
| Age | 0.97 | [0.93, 1.01] | 0.12 | 0.96 | [0.92, 1.00] | 0.068 | 0.95 | [0.91, 0.99] | 0.027 |
| Gender | 1.10 | [0.42, 2.91] | 0.8 | 1.13 | [0.42, 3.08] | 0.8 | 1.11 | [0.42, 2.90] | 0.8 |
| miRNA | 0.46 | [0.28, 0.71] | 0.001 | 1.61 | [1.27, 2.12] | 0.0002 | 1.44 | [1.15, 1.87] | 0.003 |

**Table S4: Multivariable logistic models.** For each of the 3 miRNAs, the results of a logistic regression model for case/control status including age and gender are reported. OR=Odds Ratio, CI=Confidence Interval.

**Supplementary Figure 1**: MAP2 (as possible *hsa-miR-200c-3p* target) differential expression level in normal skin and melanoma (p< 0.01). Data and analysis from Gepia2 (<http://gepia2.cancer-pku.cn/#index>)

*[1] Analysis of Relative Gene Expression Data Using Real-Time Quantitative PCR and the 2^-ΔΔCT Method* Kenneth J. Livak and Thomas D. Schmittgen. METHODS (2001)
